# Otolaryngological complications of hypopharyngeal‐esophageal multichannel intraluminal impedance‐pH monitoring

**DOI:** 10.1002/ccr3.3238

**Published:** 2020-08-15

**Authors:** Francois Bobin, Sven Saussez, Jérôme R. Lechien

**Affiliations:** ^1^ Laryngopharyngeal Reflux Study Group of Young Otolaryngologists of the International Federation of Otorhinolaryngological Societies (IFOS) Paris France; ^2^ Polyclinique Elsan de Poitiers Poitiers France; ^3^ Department of Otolaryngology‐Head Neck Surgery CHU Saint‐Pierre Université Libre de Bruxelles Brussels Belgium; ^4^ Department of Otolaryngology‐Head Neck Surgery Foch Hospital UFR Simone Veil University Versailles Saint‐Quentin‐en Yvelines (University Paris Saclay) Paris France

**Keywords:** complication, impedance, laryngopharyngeal, pH monitoring, reflux, testing

## Abstract

Probe of pH study may kink in the esophagus leading to nasal symptoms during the removal.

## Graphical abstract

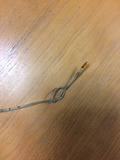

## 1 INTRODUCTION

pH Study is a routine procedure in some European laryngology offices to diagnose laryngopharyngeal reflux (LPR). Probe movements may complicate the testing leading to digestive symptoms and nose pains during the removal of the probe.

Hypopharyngeal multichannel intraluminal impedance‐pH monitoring (HEMII‐pH) with nasopharyngeal catheter placement is the standard of care for the diagnosis of gastroesophageal reflux disease (GERD) or laryngopharyngeal reflux (LPR).^1^, ^2^ The approach is safe but may be awkward for patients. In a previous study, 61% of patients reported that the test bothered them most of the time, although 69% said that they would repeat the test if needed.^3^ The placement of the HEMII‐pH probe is made in‐office and may be realized by both gastroenterologist and otolaryngologist. To date, a little number of studies reported complications of the procedure, which consisted of pain, discomfort, or nose bleeding.

The aim of this paper was to report two unusual complications of HEMII‐pH.

## 2 CASE 1

**Figure 1:**
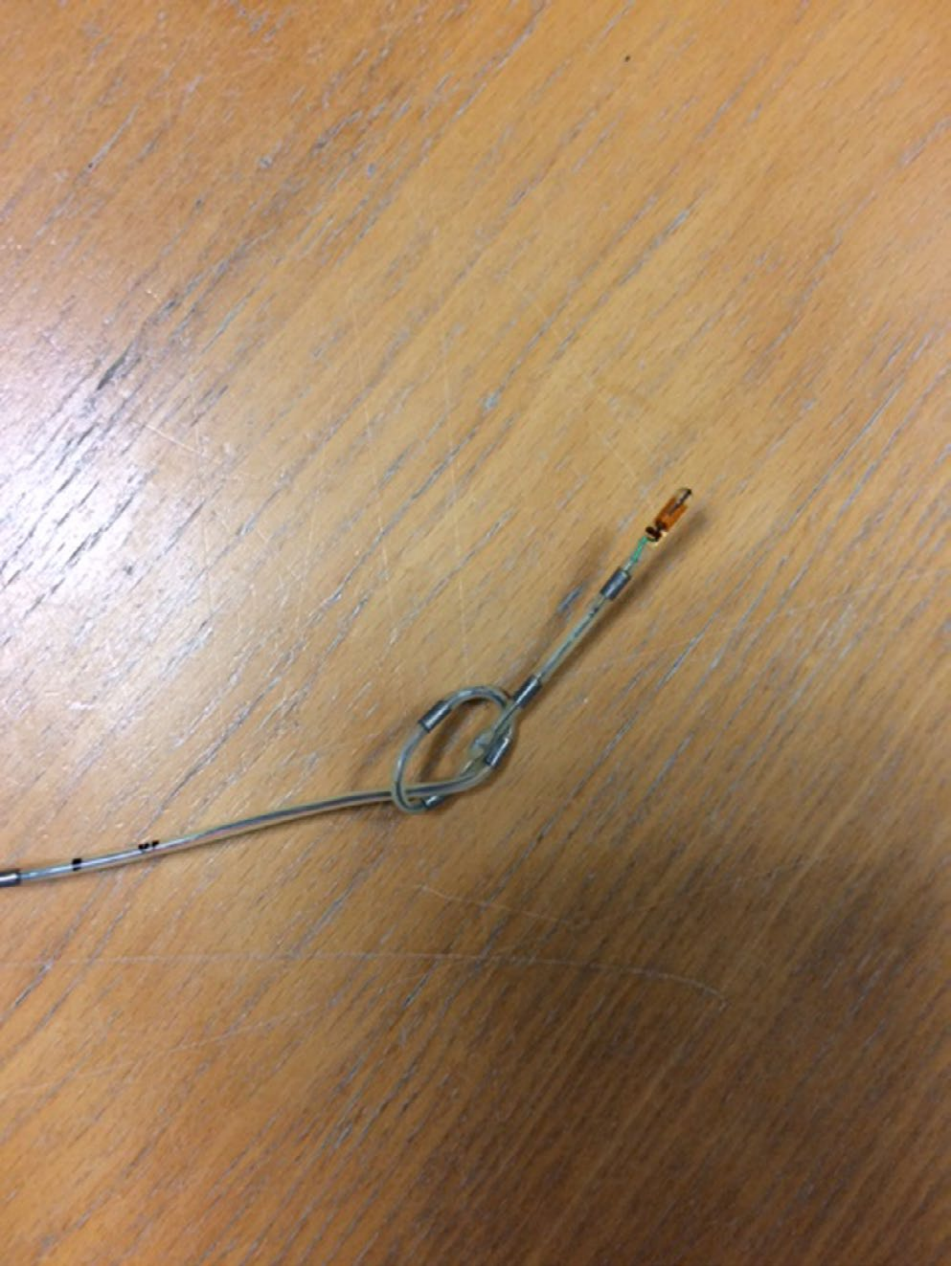
The probe node

**Figure 2:**
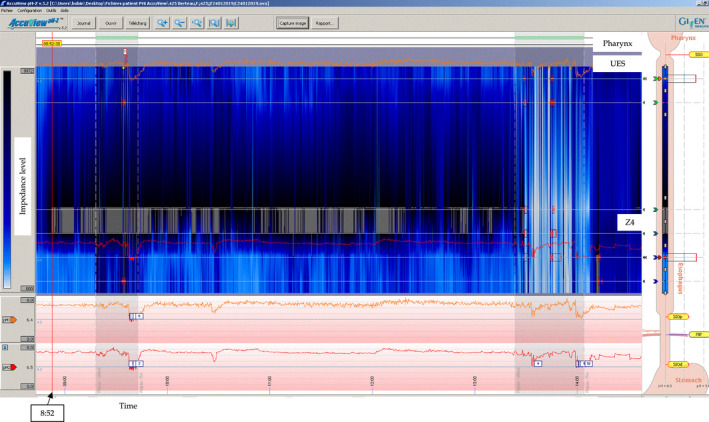
The hypopharyngeal multichannel intraluminal impedance‐pH monitoring tracing. Footnotes: Impedance is represented by a colorimetric scale from 800 ohms (white color, low impedance) to 10 000 ohms (blue color, high impedance). The gray color consists of a nonphysiological impedance value >10 000 ohms, which is a parasitic tracing. From 8:00 to 8:52, the tracing revealed gaseous reflux episodes related to belching. From 8:52, a gray area appeared at the Z4 impedance segment, which consists of the node development

A 65‐year‐old patient was referred to the Reflux Consultation of the Elsan Polyclinic (Poitiers) for a chronic history of belching, postnasal drip, dysphonia, globus, and heartburn. The nasofiberoptic examination found posterior commissure hypertrophy and arytenoid erythema. The history of the patient included refractory gastroesophageal reflux disease (GERD), while the gastrointestinal (GI) endoscopy reported Hill 2 hiatal hernia and esophagitis (grade A). Regarding the laryngopharyngeal complaints and the previous resistance to proton pump inhibitors (PPIs), a HEMII‐pH testing (VersaFlex‐Z®, Medtronic, Europe) was realized to confirm the diagnosis. The HEMII‐pH was composed of 8 impedance segments and 2 pH electrodes. The catheter model used was introduced transnasally and considered the esophageal length of patient (GI endoscopy/manometry). Six impedance segments were placed along the esophagus zones (Z1 to Z6), and they were centered at 19, 17, 11, 9, 7, and 5cm above the lower esophagus sphincter (LES). Two additional impedance segments were placed 1 and 2 cm above the upper esophagus sphincter (UES) in the hypopharyngeal cavity. The two pH electrodes were placed 2 cm above LES and 1‐2 cm below UES. The probe was fixed to an external electronic data recorder that monitors the esophageal pH. The association between symptoms and reflux episodes was studied: Patient recorded the time of meals and the occurrence of key symptoms (belching, globus, and heartburn) through the HEMII‐pH device. The patient came back 24 hours after the placement of HEMII‐pH catheter to remove it. He reported the occurrence of dysphagia 2 hours after the probe placement. The removal of the probe was associated with nose pain, and the otolaryngologist discovered a distal probe node (Figure 1). The HEMII‐pH tracing analysis showed a correct functioning of the system during the first hour of the testing period before the occurrence of several belching episodes, which were reported by the patient through the device. At this time, some parasites appeared in the tracing, reflecting the node formation (Figure 2). The rest of the recording confirmed the nonacid LPR diagnosis through the recording of the proximal probes, which were not impacted by the node. The patient consent was obtained for the publication.

## 3 CASE 2

**Figure 3:**
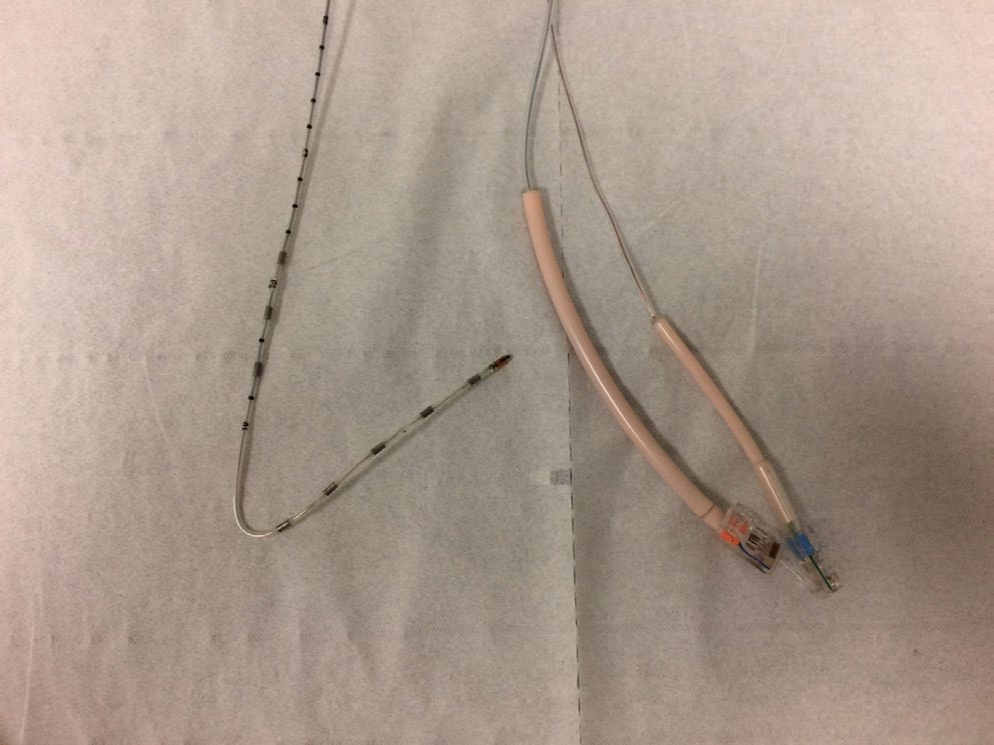
The folded probe

A 15‐year‐old patient with severe asthma was addressed to the reflux clinic to exclude ear, nose, and throat disorders that may worsen asthma. The symptoms of the patient consisted of chronic cough, throat clearing, and globus sensation. The fiberoptic examination reported edema and erythema of the posterior commissure. According to the clinical findings, HEMII‐pH was performed to exclude the LPR diagnosis. The placement of the HEMII‐pH catheter (VersaFlex‐Z®, Medtronic, Europe) and the characteristics of the device were similar to those reported in the first case. The patient had belching, nausea, and several vomiting episodes during the probe placement. During the 24‐hour testing, the patient reported many heartburn and belching episodes, which were not reported in the initial clinical picture. The removal of the probe was associated with nose pain, and the physician found a folded probe (above Z5 segment; Figure 3). Tracing revealed parasites in the distal impedance segment throughout the 24‐hour testing period. According to the tracing, the probe was probably folded during the vomiting episodes (placement time) and the related retrograde esophageal contraction. The rest of the recording confirmed the nonacid LPR diagnosis. The patient consent was obtained.

## 4 DISCUSSION

The most common complications/adverse effects of HEMII‐pH consist of nose pain, runny nose, nose bleeding, throat pain/discomfort, chest pain, cough, headache, and dysphagia.^4^ Catheter displacement is known to be a potential problem that may impair the test accuracy.^5^ The pH probe movements usually depend on the body position, bolus size and composition, and talking.^5^ During swallowing, the pH probe began by ascending 0.5‐2.0 cm and then returned to the baseline position. A study found that, for 48 swallows, the ascending oscillation was followed by a descending oscillation of up to 2.0 cm before returning to baseline; the pH probe movements could account for the variation in results and reproducibility of simultaneous esophageal pH monitoring. In this paper, we reported two rare complications of HEMII‐pH characterized by probe movements related to retrograde esophageal peristaltic events (belching and vomiting). Regarding the features of the first patient, hiatal hernia could be a favoring factor. To the best of our knowledge, this kind of complication was never reported. Otolaryngologist who performed in‐office HEMII‐pH testing has to be aware about the risk of folded probe or probe node in patients with vomiting events or belching. The removal of the probe has to be cautious in patients with a clinical suspicion of probe movements regarding the theoretical risk of nasal traumatism.

## 5 CONCLUSION

In this paper, we reported two very rare complications of HEMII‐pH, consisting of probe movements and node in the esophagus. These complications may affect the tracing analysis if the HEMII‐pH testing is realized for GERD. For LPR, the impact is lower because the LPR diagnosis is mainly based on the occurrence of hypopharyngeal reflux episodes.

## CONFLICT OF INTEREST

None declared.

## AUTHOR CONTRIBUTIONS

JRL: wrote the paper, analyzed the case, conducted literature review, and performed the research. FB and SS: analyzed the case and wrote the part: “case report.”

## INFORMED CONSENT

Patient consented to the publication.
